# Role of tissue factor in the procoagulant and antibacterial effects of human adipose-derived mesenchymal stem cells during pneumosepsis in mice

**DOI:** 10.1186/s13287-019-1391-x

**Published:** 2019-09-23

**Authors:** Desirée Perlee, Alex F. de Vos, Brendon P. Scicluna, Anja Maag, Pablo Mancheño, Olga de la Rosa, Wilfried Dalemans, Sandrine Florquin, Cornelis van’t Veer, Eleuterio Lombardo, Tom van der Poll

**Affiliations:** 1 Center of Experimental & Molecular Medicine, Amsterdam University Medical Centers, University of Amsterdam, Amsterdam, The Netherlands; 2 Department of Clinical Epidemiology, Biostatistics and Bioinformatics, Amsterdam University Medical Centers, University of Amsterdam, Amsterdam, The Netherlands; 3 TiGenix SAU, Madrid, Spain; 4 TiGenix NV, Leuven, Belgium; 5 Department of Pathology, Amsterdam University Medical Centers, University of Amsterdam, Amsterdam, The Netherlands; 6 Division of Infectious Diseases, Amsterdam University Medical Centers, University of Amsterdam, Meibergdreef 9, Room G2-130, 1105AZ Amsterdam, the Netherlands

**Keywords:** Sepsis, Mesenchymal stem cells, Immunomodulation, Coagulation

## Abstract

**Background:**

Adult mesenchymal stem cells (MSCs) improve the host response during experimental sepsis in animals. MSCs from various sources express a procoagulant activity that has been linked to the expression of tissue factor. This study sought to determine the role of tissue factor associated with adipose-derived MSCs (ASCs) in their procoagulant and antibacterial effects during pneumonia-derived sepsis.

**Methods:**

Mice were infused intravenously with ASCs or vehicle after infection with the common human pathogen *Klebsiella pneumoniae* via the airways.

**Results:**

Infusion of freshly cultured or cryopreserved ASCs induced the expression of many genes associated with tissue factor signaling and coagulation activation in the lungs. Freshly cultured and cryopreserved ASCs, as well as ASC lysates, exerted procoagulant activity in vitro as determined by a fibrin generation assay, which was almost completely inhibited by an anti-tissue factor antibody. Infusion of cryopreserved ASCs was associated with a rise in plasma thrombin-antithrombin complexes (indicative of coagulation activation) and formation of multiple thrombi in the lungs 4 h post-infusion. Preincubation of ASCs with anti-tissue factor antibody prior to infusion prevented the rise in plasma thrombin-antithrombin complex concentrations but did not influence thrombus formation in the lungs. ASCs reduced bacterial loads in the lungs and liver at 48 h after infection, which was not influenced by preincubation with anti-tissue factor antibody. At this late time point, microthrombi in the lungs were not detected anymore.

**Conclusion:**

These data indicate that ASC-associated tissue factor is responsible for systemic activation of coagulation after infusion of ASCs but not for the formation of microthrombi in the lungs or antibacterial effects.

**Electronic supplementary material:**

The online version of this article (10.1186/s13287-019-1391-x) contains supplementary material, which is available to authorized users.

## Introduction

Sepsis is a life-threatening condition characterized by a dysregulated host response to an infection resulting in organ failure [1, 2]. Even with the availability of antibiotics, sepsis remains a major health problem with an estimated worldwide population incidence rate of 437 cases per 100,000 person years [3]. Most sepsis cases are caused by pneumonia [4, 5], with *Klebsiella* (*K.*) *pneumoniae* reported as frequent causative pathogen [6, 7].

Adult mesenchymal stem cells (MSCs) could potentially be a good adjunctive therapy for sepsis. MSCs are able to secrete anti-inflammatory and antibacterial molecules and showed beneficial effects in various animal sepsis models [8–18]. Notably, however, several reports have indicated that MSCs can exert procoagulant effects [19, 20]. Infusion of MSCs in patients with complications of hematopoietic stem cell transplantation resulted in increases in the plasma concentrations of thrombin-antithrombin complexes (TATc) and d-dimer (indicative of activation of the coagulation system) in the absence of clinically evident thromboembolic events [21]. In agreement, we reported increases in the plasma levels of TATc and d-dimer in healthy humans upon infusion of adipose-derived MSCs (ASCs); ASCs in addition enhanced activation of the coagulation system elicited by intravenous injection of endotoxin in these subjects [22]. In mice, intravenous infusion of high doses of MSCs was associated with the transient formation of fibrin clots in the lungs [19]. In vitro studies have demonstrated that MSCs from several sources, including ASCs used in the aforementioned endotoxin challenge study in healthy subjects [22], express tissue factor-dependent procoagulant activity [19–25]. Considering that tissue factor has been implicated as a main initiator of coagulation [26, 27], together these data suggest that MSC-associated tissue factor is responsible, at least in part, for the procoagulant effects exerted by MSC infusion.

We recently described that infusion of ASCs was associated with reduced bacterial growth and inflammatory responses in the lung during *K. pneumoniae*-induced pneumosepsis [28]. These beneficial effects were associated with a transient formation of microthrombi in the lungs occurring shortly after the infusion of ASCs. Importantly, our group previously found that formation of fibrinogen in the lung improves host defense in this model of bacterial pneumosepsis [29], which suggests that the reduction in bacterial loads in ASCs-infused mice could be partially caused by the prothrombotic effects induced by ASCs. Therefore, in the current study we sought to (a) determine the procoagulant effects of ASCs in the lungs in more detail, (b) assess the role of ASC-associated tissue factor in the procoagulant and prothrombotic properties of ASCs, and (c) determine the contribution of ASC-associated tissue factor to the improvement of host defense by ASC infusion during pneumosepsis.

## Materials and methods

### Mice

Pathogen free 8- to 10-week-old female wild-type (WT) C57BL/6 mice were purchased from Charles River (Leiden, The Netherlands). All animals were specific pathogen-free and housed in the Animal Research Institute Amsterdam facility under standard care. All experiments were carried out in accordance with the Dutch Experiment on Animals Act and were approved by the local animal welfare committee of the Academic Medical Center. Groups consisted of *N* = 4–8 mice for each time point as indicated in the figure legends.

### Stem cell preparation

ASCs were prepared at TiGenix SAU (Madrid, Spain) as described previously [30]. In short, ASCs were obtained from adipose tissue from a healthy donor and expanded until population doubling 12–16. ASCs fulfilled the ISCT criteria for MSCs and were thoroughly checked for viability, population doublings, morphology, potency, identity, purity, sterility and genetic stability, among other quality controls. Cells were kept in liquid nitrogen until required. For the preparation of freshly cultured ASCs, cells were thawed and recovered in tissue culture flasks with Dulbecco’s modified Eagle’s medium (DMEM) supplemented with 10% fetal bovine serum (FBS), 2 mM l-glutamine, 100 units/ml penicillin, and 100 μg/ml streptomycin (37 °C at 5% CO_2_). At the day of administration, cells were trypsinized, washed in phosphate-buffered saline (PBS), and resuspended at the required concentration in Ringers lactate. Cryopreserved cells were thawed and directly resuspended at the required concentration in Ringers lactate.

### Experimental study design

Pneumonia was induced by intranasal inoculation with 10^4^ colony forming units (CFU) *K. pneumoniae* serotype 2 (ATCC 43816, Rockville, MD, USA) as previously described [28, 31–33]. At 1 or 6 h after infection, mice were intravenously infused with 1 × 10^6^ ASCs in 200 μl Ringers lactate or with 200 μl Ringers lactate (placebo). Mice infused with ASCs 1 h after infection were euthanized 4 or 16 h after induction of pneumonia; mice infused with ASCs 6 h after infection were euthanized 48 h after induction of pneumonia. Citrate-anticoagulated blood and organs were harvested and processed as described previously [31, 34].

### Microarrays and bioinformatics

For analyses of gene expression pathways related to coagulation activation, mice were infused with ASCs 1 h after infection and euthanized 16 h post infection. RNA was isolated from lung homogenate using the Nucleospin RNA isolation kit (Macherey-Nagel, Düren, Germany) as described by the manufacturer. RNA samples with integrity number (RIN) > 6 (Agilent Bioanalyzer) were included for microarrays. RNA was hybridized to the mouse Clariom S Assay HT chip (ThermoFisher Scientific) and scanned at the Cologne Center for Genomics, Cologne, Germany, as per manufacturer’s instructions. All analyses were done exactly as described previously [28]. Pathway analysis was performed by Ingenuity Pathway Analysis (IPA, Qiagen Bioinformatics) specifying the Ingenuity knowledgebase as reference set and human species. Significance was evaluated by Fisher’s exact test and Benjamini-Hochberg adjusted *p* values (adjusted *p* < 0.05). Normalized and non-normalized array data are accessible through the Gene Expression Omnibus with accession number GSE121970.

### Blocking ASC-associated tissue factor in vitro and “ex vivo”

The role of ASC-associated tissue factor in the procoagulant effect of ASCs was studied in vitro by measuring fibrin generation spectrophotometrically using the fibrin polymerization method as described [35]. In short, citrated platelet poor plasma (80 μl) was diluted in 25 mM Hepes, 137 mM NaCl, 0.1% ovalbumin (12.6 μl) with or without 25.000 freshly cultured or cryopreserved ASCs (5 × 10^6^ ASCs/ml), or ASC lysates (created by 3 freeze-thaw cycles) from the same donor in phosphate-buffered saline (5 μl), and with or without monoclonal anti-human tissue factor antibody (2.4 μl, ref. 4509, Sekisui, Stamford, CT; 10 μg/ml) in round bottom 96-well plates with Immunolon 2-high-binding coating (Fisher Scientific, Landsmeer, The Netherlands). Plasma was incubated at 37 °C for 5 min, and fibrin generation was initiated by prewarmed 25 mM Hepes, 137 mM NaCl, 0.1% ovalbumin, and 17 mM CaCl2 (20 μl). Absorption at 405 nm was measured for 60 min with 15 s intervals in the SpectraMax microplate reader (Molecular Devices, Sunnyvale, CA, USA). Results are expressed as percentage tissue factor clot activity or in arbitrary units (A.U). TF activity was determined by comparison of clot times of samples to a standard curve prepared by serial dilution of a concentrated ASC preparation. The role of ASC-associated tissue factor in the procoagulant effect of ASCs in vivo was studied by preincubating cryopreserved ASCs (5 × 10^6^ ASCs/ml) with or without monoclonal anti-human tissue factor antibody (Sekisui, Stamford, CT; 50 μg/ml) for 30 min prior to infusion; in mice not receiving ASCs, vehicle was preincubated with anti-tissue factor antibody. In these experiments, mice were infused with ASCs or vehicle 1 or 6 h after infection and euthanized at 4 or 48 h respectively. In all conditions wherein the effect of anti-tissue factor was tested, control samples were treated with an isotype control antibody (LEAF purified mouse IgG1 κ, Biolegend, San Diego, CA; respectively 10 or 50 μg/ml).

### Assays

TATc was measured by ELISA (Affinity Biologicals, Ancaster, Ontario, Canada) in plasma following the manufacturer’s instructions.

### Histology and immunohistochemistry

Lungs were harvested, fixed in 10% formaldehyde, and embedded in paraffin. Four-micrometer sections were stained with hematoxylin and eosin (H&E) and scored as described [31].

### Statistical analysis

Data are expressed as box and whisker plots or as bars (mean with SD). Comparisons between multiple groups were first performed using Kruskal-Wallis one-way analysis of variance test, followed by Mann-Whitney *U* test where appropriate. Analysis was done using GraphPad Prism version 6.0 (Graphpad Software, San Diego, CA). *p* values < 0.05 were considered statistically significant.

## Results

### Adipose-derived mesenchymal stem cells induce procoagulant gene expression in the lungs

**Fig. 1:**
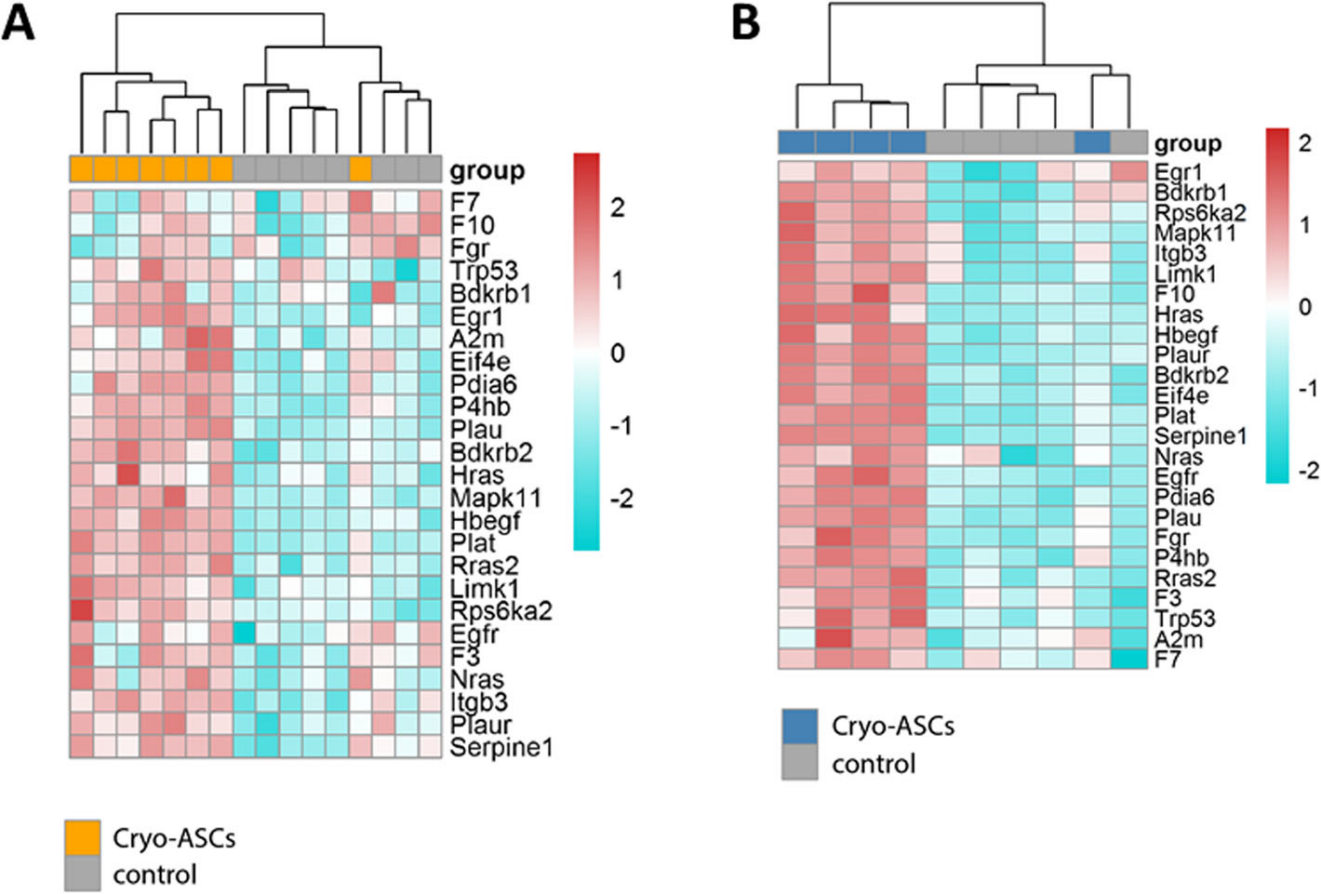
Infusion of ASCs induce expression of genes associated with coagulation in the lung. Mice were infused with 1 × 10^6^ cryopreserved ASCs and lungs were harvested 15 h thereafter. Genes associated with coagulation by Ingenuity Pathway Analysis that were significantly altered by infusion of cryopreserved ASCs in naïve mice (**a**) and mice infected with *Klebsiella pneumoniae* via the airways 1 h prior to ASC infusion (**b**). Heatmap representation of transcript expression (rows) in control and ASC treated animals. Red, high expression; turquoise, low expression

We previously reported that intravenous infusion of ASCs induces transient thrombus formation in the lungs after treatment in mice [28]. We here wished to establish whether infusion of freshly cultured and/or cryopreserved ASCs elicited expression of genes associated with activation of coagulation in the lungs. For this, we evaluated genome-wide mRNA expression profiles in whole lungs obtained from mice that were infused with freshly cultured or cryopreserved ASCs (or vehicle only) 15 h earlier for significantly altered gene expression pathways using IPA. Indeed, compared with vehicle control both freshly cultured and cryopreserved ASCs significantly upregulated the IPA pathways “Role of tissue factor in cancer” and “Coagulation system” in uninfected mice. Figure 1a shows the heatmap of genes listed in these pathways that were significantly altered upon infusion of cryopreserved ASCs. To determine the effect of ASCs on coagulation-related gene expression during pneumonia, we infected mice with the common human pathogen *K. pneumoniae* via the airways 1-h prior to ASC infusion (thereby mimicking a therapeutic setting) and analyzed mRNA profiles 16 h later (i.e., 15 h after ASC administration). In mice infused with vehicle control infection, with *Klebsiella* was associated with an upregulation of the IPA pathways “Role of tissue factor in cancer” and “Coagulation system” in the lungs (data not shown). While freshly cultured ASCs did not significantly modify infection-induced upregulation of coagulation-related genes, cryopreserved ASCs further enhanced gene expression in the IPA pathway “Role of tissue factor in cancer” in infected mice (Fig. 1b).

### ASCs induce tissue factor-dependent procoagulant activity in vitro

**Fig. 2:**
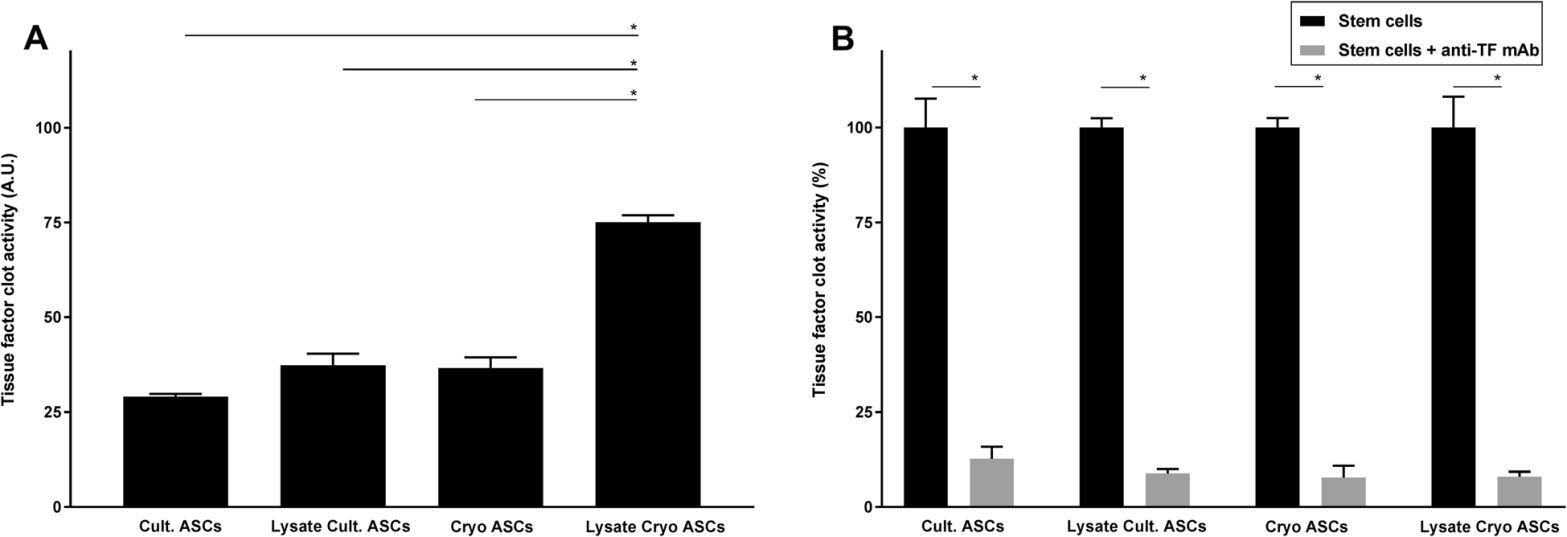
Intact ASCs and ASC lysates exert procoagulant activity in vitro. In vitro fibrin generation in normal pool plasma by cultured and cryopreserved ASCs and ASC lysates shown in arbitrary units (**a**). Tissue factor dependence is shown by an anti-tissue factor antibody (**b**). Data are expressed as mean (with SD) tissue factor clot activity (A.U. or %) of ASCs and lysates derived from 1 donors (*N* = 4). *p < 0.05

We previously reported that freshly cultured ASCs express tissue factor-dependent procoagulant activity in vitro [22], which is in agreement with several earlier reports [19–21, 23–25]. Here we show that cryopreserved ASCs and ASC lysates also express procoagulant activity, using a fibrin generation assay (Fig. 2a). Procoagulant activities of intact ASCs or ASC lysates could be blocked almost completely by the addition of an anti-human tissue factor antibody (Fig. 2b).

### Blocking tissue factor on ASCs prior to infusion inhibits systemic activation of coagulation but not thrombus formation in the lungs

**Fig. 3:**
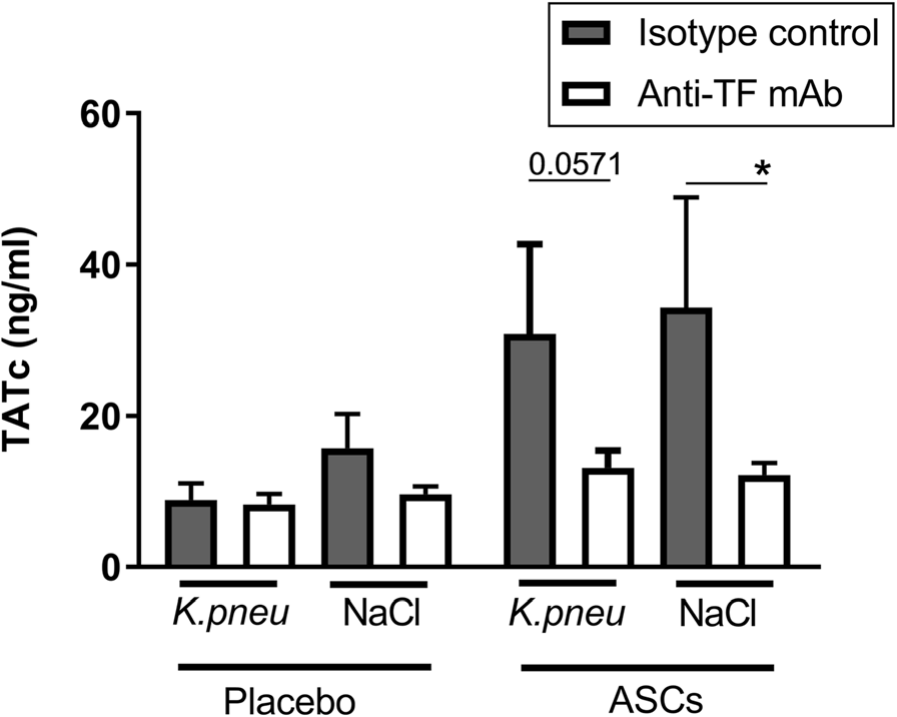
Activation of the coagulation system induced by intravenous ASCs is dependent on ASC-associated tissue factor. Plasma concentrations of thrombin-antithrombin complexes (TATc) 3 h after intravenous infusion of 1 × 10^6^ cryopreserved ASCs measured in uninfected mice and mice infected with *K. pneumoniae* via the airways 1 h prior to ASC infusion. ASCs were preincubated with a blocking anti-tissue factor antibody or isotype control prior to infusion. Data are expressed as bars (mean with SD), *N* = 4 mice per group. *p < 0.05 versus the control group

**Fig. 4:**
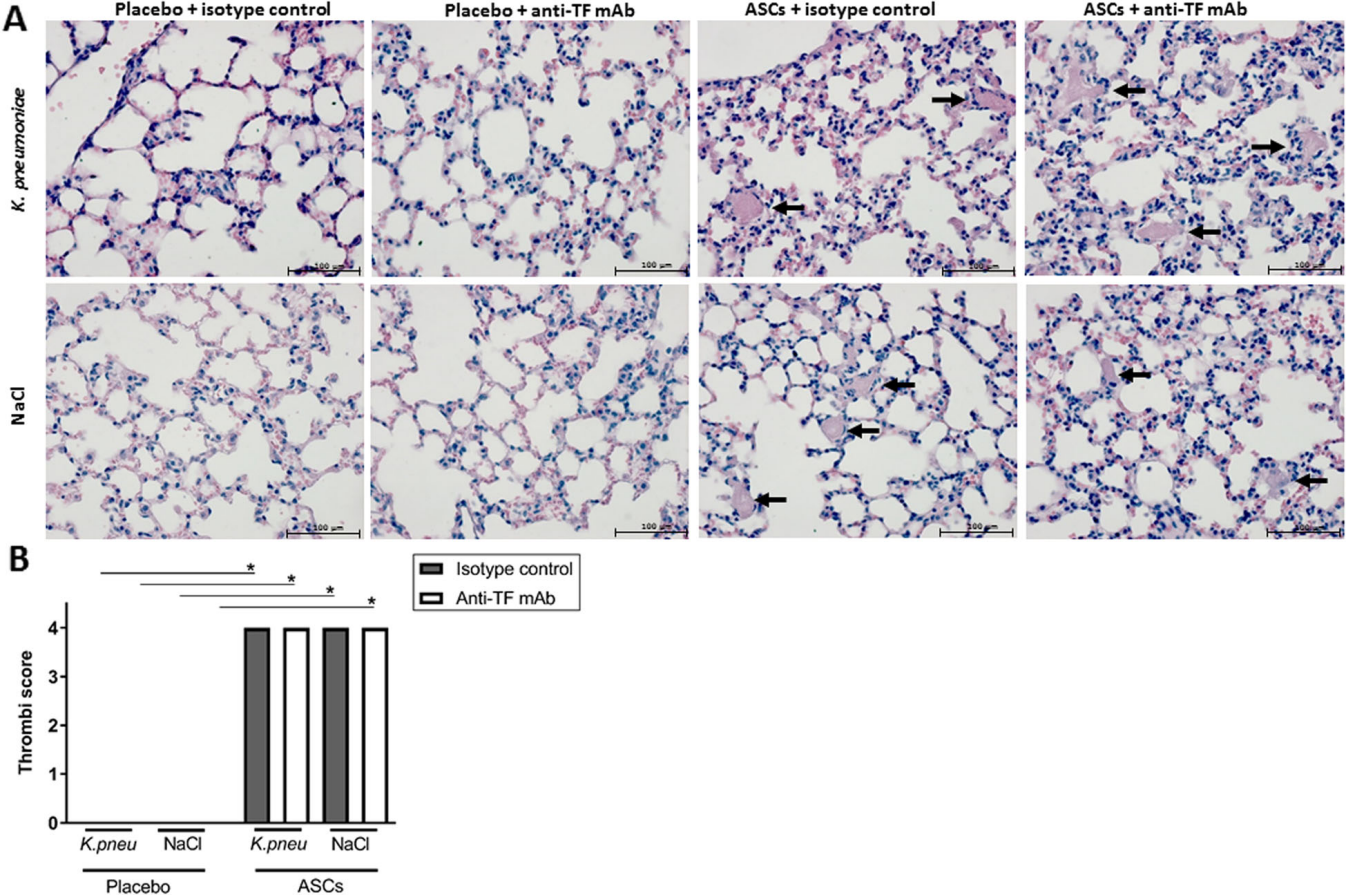
ASC infusion results in thrombi formation in the lungs which is not affected by preincubation with an anti-tissue factor antibody. Mice were treated with 1 × 10^6^ cryopreserved ASCs intravenously and lungs were harvested 3 h thereafter. Some mice were infected with *K. pneumoniae* via the airways 1 h prior to ASC infusion. ASCs were preincubated with a blocking anti-tissue factor antibody or isotype control prior to infusion. **a** Representative photographs of H&E-stained tissue sections of infected and uninfected lungs at 4 h, showing thrombi formation in ASCs-treated mice (arrows); original magnification × 40. **b** Quantification of thrombus formation (0 = no thrombi, 1 = 1 thrombus, 2 = 2–5 thrombi, 3 = 6–10 thrombi, 4 = > 10 thrombi per microscopic section). Data are expressed as bars for panel **b** (mean with SD). *N* = 4 mice per group. *p < 0.05 versus the control group

We previously showed that intravenous infusion of ASCs into healthy humans results in a brisk activation of the coagulation system as reflected by a rapid rise in plasma TATc levels [22]. We here wished to determine whether this in vivo response is tissue factor dependent. For this, we preincubated the clinically relevant cryopreserved ASCs with the blocking anti-human tissue factor antibody also used in the experiments shown in Fig. 2b prior to intravenous infusion into mice. We used a fivefold higher concentration of the anti-tissue factor antibody as used in vitro to ascertain blocking of ASC-related tissue factor in vivo. Like in healthy humans [22], intravenous infusion of ASCs induced an increase in plasma TATc levels early after administration (4 h), which was detectable in both uninfected mice and mice infected with *Klebsiella* via the airways (Fig. 3). Importantly, preincubation of ASCs with the anti-tissue factor antibody prevented the increase in plasma TATc, suggesting that activation of the coagulation system by intravenous ASCs is dependent on ASC-associated tissue factor. Infusion of ASCs was associated with the formation of multiple small thrombi in the lungs, confirming our previous data [28] and those of others [19] (Fig. 4). This prothrombotic response was not influenced by preincubation of ASCs with anti-tissue factor, suggesting that thrombus formation induced by intravenous ASCs does not depend on tissue factor-dependent coagulation. ASCs also elicited an inflammatory response in the lungs as determined 4 h post infusion in both uninfected and infected mice; this was not influenced by preincubation of ASCs with anti-tissue factor (Additional file 1: Figure S1). At this early time point, ASCs did not influence bacterial loads in the lungs (Additional file 2: Figure S2; other body sites still culture negative), which is in agreement with our previous investigation demonstrating that the antibacterial effect of ASCs is detected late during the infection [28].

### Blocking tissue factor on ASCs prior to infusion does not alter their capacity to reduce bacterial loads during pneumonia-derived sepsis

**Fig. 5:**
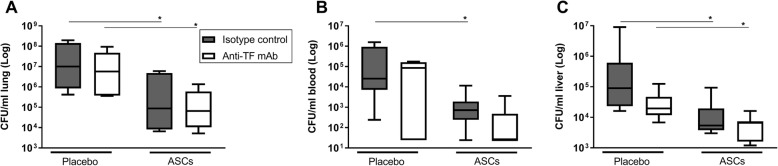
**a**–**c** ASC infusion reduces bacterial loads which is not affected by preincubation with an anti-tissue factor antibody. Bacterial loads (colony-forming units (CFUs)) in the lung, blood, and liver 48 h after infection with *K. pneumoniae* via the airways in mice treated with 1 × 10^6^ cryopreserved ASCs intravenously 6 h after bacterial inoculation. ASCs were preincubated with a blocking anti-tissue factor antibody or isotype control antibody prior to infusion. Data are expressed as box-and-whisker diagrams, *N* = 8 mice per group. *p < 0.05 versus the control group

The term immunothrombosis has been introduced to indicate that local thrombus formation may improve host defense against infection [36]. In agreement, our group recently showed that inhibition of thrombin activity or fibrin generation is associated with enhanced bacterial growth and dissemination in this model of *Klebsiella* pneumosepsis [29]. Thus, we considered it of importance to determine whether the procoagulant effects of ASCs contribute to their antibacterial properties in vivo. To this end, we preincubated ASCs with anti-tissue factor antibody prior to infusion 6 h after infection with *Klebsiella* via the airways; bacterial burdens were subsequently determined 48 h after infection. This treatment schedule and endpoint were chosen because we previously showed that ASCs infused 6 h after infection (mimicking a therapeutic setting) strongly reduced bacterial loads at 48 h post infection [28]. As shown in Fig. 5, ASC reduced bacterial loads in lungs, blood, and liver, which was not influenced by preincubation with the anti-human tissue factor antibody. At this late time point, lungs did not show microthrombi in any treatment group (data not shown), consistent with our earlier study reporting that thrombus formation occurs early after ASC infusion and is transient [28]. In addition, plasma TATc levels were lower than those detected early after infusion of ASCs and not different between groups (Additional file 3: Figure S3), confirming the transient nature of the effect of ASCs on the coagulation system.

## Discussion

MSCs improve a variety of outcome parameters, including survival, in preclinical sepsis models [8–18] and are currently evaluated in clinical trials in critically ill patients with sepsis and/or acute respiratory distress syndrome [37, 38]. Additional research is focused on the mechanism of action and on possible side effects of MSC therapy in this context. One of these side effects repeatedly reported in both animals [19, 20] and humans [21, 22] is the procoagulant effect of MSCs. Even though MSCs administration was proven to be safe in multiple clinical trials [37, 38], their procoagulant effect needs to be explored as the accumulation of MSCs could possibly result in microvascular thrombosis [19, 25, 28]. In addition, it is conceivable that the procoagulant properties of MSCs contribute to their bacterial growth limiting capacity, especially at local sites of infection such as the lung [28, 29, 36]. Thus, considering that MSC-associated tissue factor has been implicated as a potential driver of coagulation activation by MSCs [19–25], we sought to determine the role of cell-associated tissue factor in the procoagulant effects of ASCs in *Klebsiella pneumoniae* induced pneumosepsis, as well as its contribution to restricting bacterial growth herein. Our main findings indicate that while ASC-associated tissue factor is responsible for systemic activation of the coagulation system upon infusion of ASCs, it does not contribute to the transient occurrence of microthrombi in the lung or to the antibacterial effects.

We analyzed the expression of genes linked to tissue factor function and coagulation activation in the lungs of mice exposed to ASCs. Multiple genes directly involved in coagulation (*F3*, *F7*, *F10*, encoding tissue factor, clotting factors VII and X respectively) were induced by ASCs in lungs of uninfected mice, together with genes associated with related host response systems, such as the fibrinolytic system (*Plaur*, *Plat* and *Serpine-1*, encoding urokinase receptor, tissue-type plasminogen activator and plasminogen activator inhibitor type 1 respectively) and the kininogen-kinin system (*Bdkrb1* and *Bdkrb2*, encoding bradykinin receptors 1 and 2). Cryopreserved ASCs further upregulated most of these genes during *Klebsiella* pneumonia. These data, together with our previous report on genome-wide gene expression in mice infused with ASCs [28], indicate that ASCs exert strong and broad effects in lung tissue upon intravenous administration, which includes a set of coagulation-related genes. Cryopreserved ASCs had a stronger effect on coagulation-related gene expression than freshly cultured ASCs. The findings that blocking of tissue factor abrogated tissue factor expressed by cryopreserved ASCs in vitro, as well as activation of coagulation in vivo, suggests that the difference between cryopreserved and freshly cultured ASCs may relate to differential activity of tissue factor. Clearly, further studies are needed to address this issue. Notably, RNA expression profiles reported relate to mouse tissue since a mouse-specific micro-array was used. We were not successful in obtaining sufficient ASCs from mouse lungs for analyses of (human) RNA expression in these cells.

Our studies focused on the role of ASC-associated tissue factor in the activation of coagulation and thrombus formation. Tissue factor is the most important initiator of coagulation in sepsis [39]. This transmembrane receptor is normally segregated from the bloodstream, but expressed by activated macrophages/monocytes and endothelial cells and by micro particles formed by these cells [39, 40]. We here showed that not only intact ASCs but also ASCs lysates express tissue factor-dependent procoagulant activity in vitro, as determined in a fibrin generation assay. In agreement, a recent study showed that microvesicles of MSCs exert procoagulant effects in vitro [41]. To our knowledge, the effects of MSC-derived microvesicles on coagulation in vivo have not been studied thus far.

We used ASCs from a single donor. We previously showed that ASCs from different donors may (modestly) differ in the extent of tissue factor expression [22]. We studied the effects of ASCs infused up to 6 h after infection. In preliminary experiments, ASCs infused 30 h after infection did not modify bacterial growth or inflammatory responses in this model (data not shown). Notably, the *Klebsiella* strain used is highly virulent and airway infection in mice is associated with 100% mortality starting from 48 h after infection [29, 42]. In a clinical setting, ASCs will be administered in the context of a more advanced infection. As such, while the present data provide insight into the mechanism by which ASCs exert procoagulant effects in vivo, extrapolation to human sepsis should be done with caution.

Infusion of ASCs induced a rise in plasma TATc levels, indicative of activation of the coagulation system, in both naïve and infected mice. Similarly, intravenous administration of ASCs induced an increase in plasma TATc in healthy subjects [22] and bone marrow-derived MSCs elicited plasma TATc elevations in patients treated for complications of hematopoietic stem cell transplantation [21]. The current results strongly suggest that this systemic activation of the coagulation system is mediated by ASC-associated tissue factor. Indeed, preincubation of ASCs with a blocking anti-tissue factor antibody prevented the increase in plasma TATc levels after infusion of ASCs. Notably, however, in spite of this clear anticoagulant effect, anti-tissue factor treatment of ASCs prior to infusion did not influence the extent of thrombus formation in the lungs. This could have several not mutually exclusive explanations. First, the anti-tissue factor antibody did not totally block fibrin generation ex vivo, which could be related to incomplete blocking of tissue factor activity and/or to procoagulant activity independent of tissue factor. With respect to the latter option, a recent paper found evidence for an additional tissue factor-independent procoagulant mechanism in MSCs, although this was not studied in ASCs [43]. Second, ASCs could induce transient microthrombi because they get trapped in the microvasculature of the lung independent of coagulation activation. In this regard, ASCs did induce endogenous mouse TF expression in the lung (see Fig. 1a, b, “F3”), which is not blocked by the anti-human tissue factor antibody. Studies with genetically modified ASCs lacking tissue factor would be of great value to further investigate the role of ASC-associated tissue factor in the activation of coagulation and thrombus formation. We focused our studies on the effect of anti-tissue factor at early time points after ASC infusion, since we previously established that ASC infusion is associated with an early transient rise in plasma TATc levels [22] as well as microthrombi formation in the lungs [28]. The present study confirmed that microthrombi could not be detected anymore at 48 h after infection. In addition, at this late time point, plasma TATc levels were lower than those measured early after infusion of ASCs and not influenced by either ASC infusion or preincubation of ASCs with anti-tissue factor, again indicating that the effect of ASCs on the coagulation system is transient. It remains to be established whether MSCs induce microthrombi formation in humans. Regarding this, it is important to emphasize that infusion of MSCs has not been associated with thromboembolic events in patients.

Local activation of coagulation, at the site of infection, may improve host defense against bacteria [28, 29, 36]. Our study does not provide a definite answer to the question whether local thrombus formation shortly after ASC infusion contributes to the limiting effect of ASCs on bacterial growth, since anti-tissue factor treatment of ASCs prior to infusion did not modify this. Of note, MSCs can modify the host immune response in a variety of ways that may improve bacterial clearance and in addition exert direct antibacterial effects [8, 9, 16, 18].

## Conclusion

In summary, we showed that while ASC-associated tissue factor is responsible for systemic activation of the coagulation system upon intravenous infusion of ASCs, it does not influence microvascular thrombus formation in the lungs or antibacterial effects after ASC administration.

## Additional files

Additional file 1: **Figure S1**. ASCs infusion results in an increased inflammatory response in the lungs in uninfected and infected mice. Mice were treated with 1 × 10^6^ cryopreserved ASCs intravenously and lungs were harvested 3 hours thereafter. Some mice were infected with *K. pneumoniae* via the airways one hour prior to ASC infusion. ASCs were preincubated with a blocking anti-tissue factor antibody or isotype control prior to infusion. (A) Representative photographs of H&E-stained tissue sections of infected and uninfected lungs at 4 hours; original magnification 10x. (B) The extent of inflammation scored on H&E tissue sections as total HE score. Data are expressed as bars for panel B (mean with SD). *N* = 4 mice per group. * *p* < 0.05 versus the control group. (TIF 3283 kb)

Additional file 2: **Figure S2.** Effect of ASCs infusion on bacterial loads early after infection. Bacterial loads (colony-forming units (CFU’s)) in the lung, blood and liver 4 hours after infection with *K. pneumoniae* via the airways in mice treated with 1 × 10^6^ cryopreserved ASCs intravenously one hour after bacterial inoculation. ASCs were preincubated with a blocking anti-tissue factor antibody or isotype control antibody prior to infusion. Data are expressed as box-and-whisker diagrams, N = 4 mice per group. Differences between groups were not significant. (TIF 77997 kb)

Additional file 3: **Figure S3.** Activation of the coagulation system induced by intravenous ASCs. Plasma concentrations of thrombin-antithrombin complexes (TATc) 48 hours after infection with *K. pneumoniae* via the airways in mice treated with 1 × 10^6^ cryopreserved ASCs intravenously 6 hours after bacterial inoculation. ASC were preincubated with a blocking anti-tissue factor antibody or isotype control prior to infusion. Data are expressed as bars (mean with SD), *N* = 8 mice per group. Differences between groups were not significant. (TIF 1337 kb)

## Data Availability

The majority of the data generated or analyzed during this study are included in this published article [and its supplementary information files]. The datasets used and/or analyzed during the current study, and not available in this article, are available from the corresponding author on reasonable request.
