# Supplementary figures and images for: Role of tissue factor in the procoagulant and antibacterial effects of human adipose-derived mesenchymal stem cells during pneumosepsis in mice
Source: Stem Cell Res Ther. 2019 Sep 23;10:286. doi: 10.1186/s13287-019-1391-x (PMC6757441; doi:10.1186/s13287-019-1391-x)

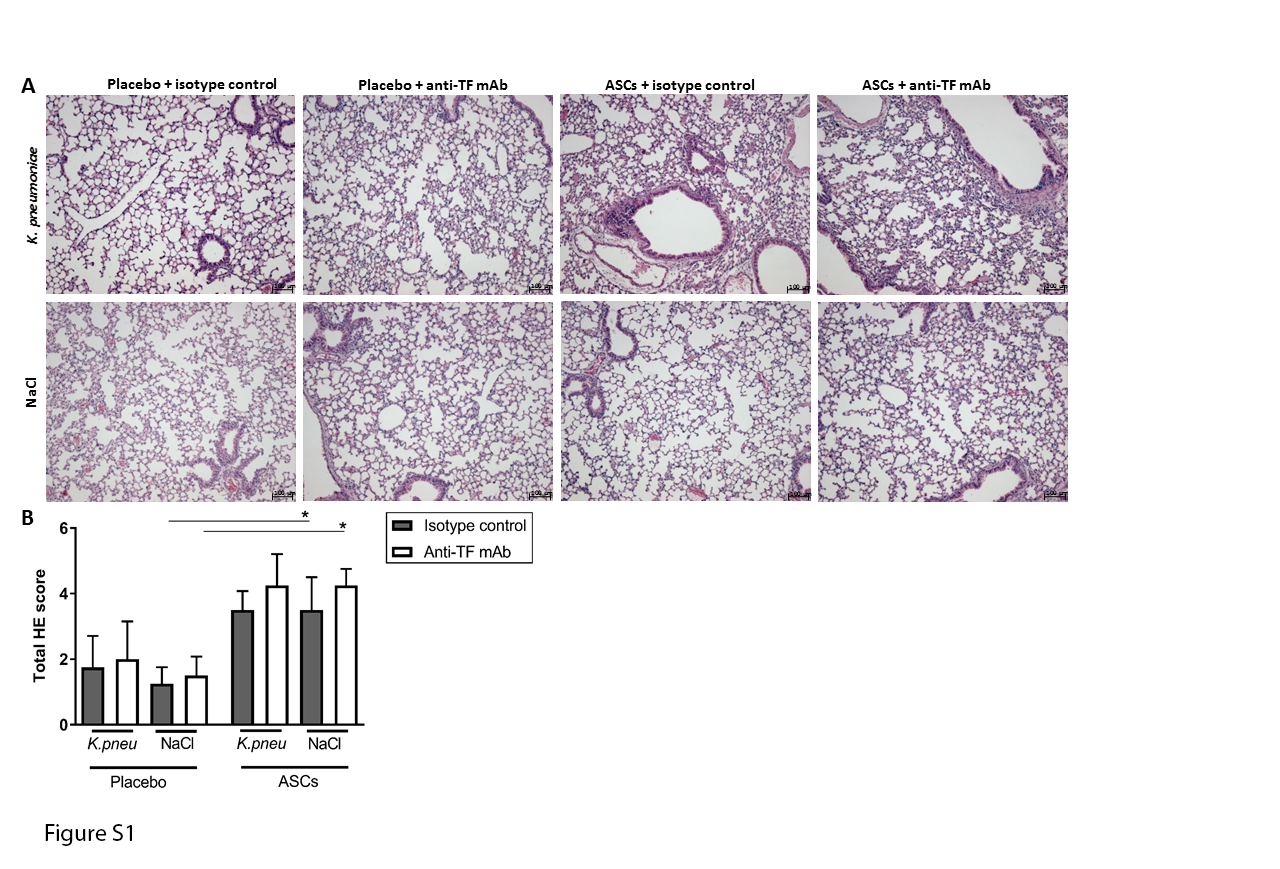

Supplement: Supplementary file 1 — Figure S1. ASCs infusion results in an increased inflammatory response in the lungs in uninfected and infected mice. Mice were treated with 1 × 10^6^ cryopreserved ASCs intravenously and lungs were harvested 3 hours thereafter. Some mice were infected with K. pneumoniae via the airways one hour prior to ASC infusion. ASCs were preincubated with a blocking anti-tissue factor antibody or isotype control prior to infusion. (A) Representative photographs of H&E-stained tissue sections of infected and uninfected lungs at 4 hours; original magnification 10x. (B) The extent of inflammation scored on H&E tissue sections as total HE score. Data are expressed as bars for panel B (mean with SD). N = 4 mice per group. * p < 0.05 versus the control group. (TIF 3283 kb) [file 13287_2019_1391_MOESM1_ESM.tif]

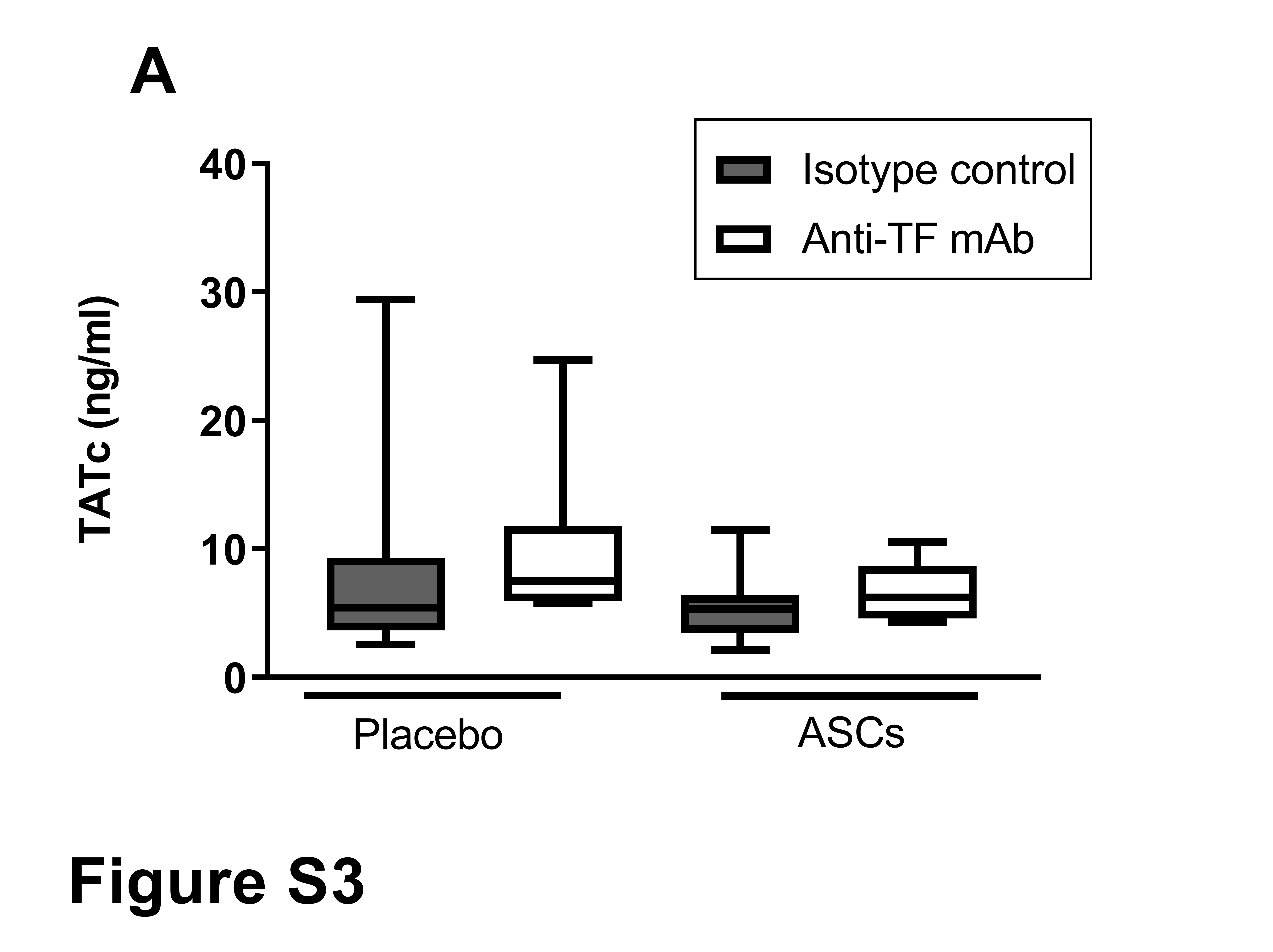

Supplement: Supplementary file 3 — Figure S3. Activation of the coagulation system induced by intravenous ASCs. Plasma concentrations of thrombin-antithrombin complexes (TATc) 48 hours after infection with K. pneumoniae via the airways in mice treated with 1 × 10^6^ cryopreserved ASCs intravenously 6 hours after bacterial inoculation. ASC were preincubated with a blocking anti-tissue factor antibody or isotype control prior to infusion. Data are expressed as bars (mean with SD), N = 8 mice per group. Differences between groups were not significant. (TIF 1337 kb) [file 13287_2019_1391_MOESM3_ESM.tif]
